# Predicting Actual Social Skill Expression from Personality and Skill Self-Concepts

**DOI:** 10.3390/jintelligence10030048

**Published:** 2022-07-29

**Authors:** Simon M. Breil, Ina Mielke, Helmut Ahrens, Thomas Geldmacher, Janina Sensmeier, Bernhard Marschall, Mitja D. Back

**Affiliations:** 1 Department of Psychology, University of Münster, 48149 Münster, Germany; mitja.back@wwu.de; 2 Department of Biochemistry and Molecular Cell Biology, University Medical Center Hamburg-Eppendorf, 20246 Hamburg, Germany; i.mielke@uke.de; 3 Department of Medicine, University of Münster, 48149 Münster, Germany; helmut.ahrens@uni-muenster.de (H.A.); thomas.geldmacher@uni-muenster.de (T.G.); janina.sensmeier@uni-muenster.de (J.S.); bernhard.marschall@ukmuenster.de (B.M.)

**Keywords:** noncognitive skills, socioemotional skills, personality traits, behavioral expression, self-other agreement

## Abstract

Social skills are of key importance in everyday and work life. However, the way in which they are typically assessed via self-report questionnaires has one potential downside; self-reports assess individuals’ global self-concepts, which do not necessarily reflect individuals’ actual social behaviors. In this research, we aimed to investigate how self-concepts assessed via questionnaires relate to skill expression assessed via behavioral observations after short interpersonal simulations. For this, we used an alternative behavior-based skill assessment approach designed to capture expressions of predefined social skills. Self- and observer ratings were collected to assess three different social skills: agency (i.e., getting ahead in social situations), communion (i.e., getting along in social situations), and interpersonal resilience (i.e., staying calm in social situations). We explored how these skills were related to self-concepts by differentiating between a classic personality measure (i.e., Big Five Inventory 2; BFI-2) and a novel skill questionnaire (i.e., Behavioral, Emotional, and Social Skills Inventory; BESSI). The results (*N* = 137) showed that both personality and skill self-concepts predicted self-rated skill expression, with the BESSI showing incremental validity. For both personality and skills self-concepts, the relationships with observer-rated skill expression were significant for agency but not for communion or interpersonal resilience. We discuss these results and highlight the theoretical and practical importance of differentiating between skill self-concepts and actual skill expression.

## 1. Introduction

People differ in how effectively they deal with complex social situations. That is, some individuals are more successful at persuading, showing compassion, or calmly dealing with criticism than others. Differences in such social skills have frequently been discussed as key person variables related to pivotal outcomes, such as academic success and job performance (Arthur et al. 2003; Ferris et al. 2001; Lievens and Sackett 2012; Soto et al. 2022).

Although there is widespread agreement on the importance of social skills, there is a lack of clarity on how to capture them. Most commonly, social skills are assessed via global self-reports that are similar (or even identical) to classic personality questionnaires (e.g., Primi et al. 2016; Riggio 1986). This enables an economical and straightforward measurement of a variety of different skills. Such global personality or skill self-reports have also been shown to predict a range of important outcome criteria (e.g., grades, salary, longevity; Chapman et al. 2011; Soto et al. 2022; Spurk and Abele 2011). However, this kind of assessment has one potential drawback: Typical rating scales assess individuals’ global skill self-concepts, which are inherently prone to biases due to motivational and informational limits (Back and Egloff 2009; Paulhus and Vazire 2007). For example, individuals may desire to maintain a positive self-concept. They might also have limited access to or knowledge about their actual social repertoire. That is, questionnaires provide insight into what individuals say they can do but not necessarily into what they can actually do (Abrahams et al. 2019).

A complement or alternative to the use of self-concept questionnaires is to rely on actual behavioral measures of skills. Given that, conceptually, social skills have an exclusive focus on the behaviors expressed in social situations, it is striking that little is known about the relationships between questionnaire-based assessments and actual expressions of social skills. Only by knowing the extent to which skill self-concepts relate to behavioral skill expression can one guide researchers’ and practitioners’ assessment decisions (see Soto et al. 2021). With this research, we aim to investigate these relationships in more detail. That is, we use an alternative behavior-based skill assessment approach. Here, various short interpersonal simulations (i.e., interactions with professional actors) are employed to capture actual expressions of predefined social skills (i.e., one simulation per skill). We explore the relationships with questionnaire-based assessments by differentiating between (a) classic personality and novel skill questionnaires, (b) self- and observer-rated expressed skills, and (c) different social skill dimensions.

### 1.1. Differentiating Skills from Personality

Social skills—often used synonymously with the terms interpersonal skills, people skills, and social competencies (Klein et al. 2006)—refer to the entire range of skills that promote effective functioning in interpersonal situations (e.g., compassion, assertiveness, patience; Breil et al. 2022a).^1^ Social skills are generally considered relatively stable over the short term but changeable over the long term through training and practice (Soto et al. 2022). This developmental potential is also what differentiates social skills from abilities (e.g., cognitive ability), which are considered mostly inherent. Individuals high in specific social skills are able to recognize that specific behaviors are beneficial in *certain* situations and are able to act accordingly (i.e., when a situation calls for it; Lechner et al. 2022). Thus, the concept of social skills can also be distinguished from personality because personality refers to typical performance (what someone *tends to do in general*), whereas skills refer to maximum performance (what someone *is capable of doing*). That is, even though both personality traits and social skills can be considered cross-situational, they differ in the type of situations in which respective individual differences become visible. For example, individuals with a high level of compassion skill do not necessarily behave more compassionately on average than others, but they are able to show compassion in situations when it matters.

Although the distinction between social skills and personality has been emphasized in recent reviews and research on social skills (Breil et al. 2022a; Danner et al. 2021; Duckworth and Yeager 2015; Napolitano et al. 2021; Soto et al. 2021; Soto et al. 2022), social skills have usually been assessed with questionnaires that aim to capture typical performance (e.g., Argyle and Lu 1990; Ferris et al. 2001; Kanning 2009b; Lechner et al. 2019; Primi et al. 2016; Riggio 1986). However, we believe that the distinction is crucial because an individual’s typical performance is not the same as their maximum performance (e.g., Klehe et al. 2014; Ployhart et al. 2001; Sackett et al. 1988; Turner 1978), and just because a person tends to do something or likes doing something does not mean that they are successful at it. Hence, personality questionnaires may assess individual differences that are strongly related to skills, but they do not directly measure skills.

Building on similar arguments, Soto et al. (2022) recently created the Behavioral, Emotional, and Social Skills Inventory (BESSI), a questionnaire that assesses skills as capacities (i.e., as maximum performance; see Buhrmester et al. 1988, for a similar approach). Here, participants are specifically asked to indicate “*how well you do that thing*,” which is clearly different from the usual classic personality questionnaire instructions that ask people to indicate, for example, “*I am someone who.*” Soto et al.’s results showed that although the proposed skills converged substantially with personality traits (i.e., an average correlation of .76 between BESSI skill domains and related BFI-2 domains), the skill domains were able to capture unique information. Nevertheless, the BESSI does not directly capture how well people perform their respective behaviors. To date, there have been no studies on how such novel skill questionnaires fare in predicting actual skill expression in comparison with classic personality questionnaires. 

### 1.2. Differentiating Self-Rated and Observer-Rated Skill Expression

Generally, only a few studies have attempted to measure specific social skills using behavioral observations (for overviews, see Abrahams et al. 2019; Duckworth and Yeager 2015). Such observations necessarily involve a systematic consideration of individuals’ expressed behavior in real or simulated social situations, calling for specific behavioral responses. This behavior would then be assessed either by the individuals themselves (which from here on we will refer to as “self-rated skill expression”) or by others (i.e., “observer-rated skill expression”). In contrast to classic questionnaires, ratings of skill expression should theoretically be less prone to retrospective biases and informational limits (because behavior is evaluated directly after a social situation). Furthermore, *observer-rated* skill expression should not be influenced by motivational limits because independent observers have no desire to portray participants in an overly positive or negative way.

For personality (i.e., typical performance), multiple studies have investigated the relationship between global self-concepts and expressed personality. For example, in experience sampling studies, personality self-concepts have been found to be moderately related to (aggregated) self-rated personality expression (*r* = .20 to *r* = .60; Finnigan and Vazire 2018; Fleeson 2009; Rauthmann et al. 2019; Sherman et al. 2015) and, to a lesser extent, to observer-rated personality expression (*r* = .01 to *r* = .36; Breil et al. 2022c). The relationships between observer-rated personality expression and self-concepts have also been investigated as part of laboratory studies (i.e., by observing behavior in predefined social situations, e.g., Back et al. 2009; Borkenau et al. 2004; Leikas et al. 2012). The results have shown that individuals have some insights into their actual behaviors, but relationships have varied and have been far from perfect (i.e., *r* = .10 to *r* = .50). 

Regarding the relationships between the expressions of specific social skills and individuals’ self-concepts, there are surprisingly few empirical findings. As one notable exception, Brackett et al. (2006) showed that observer ratings of social competence were not related to individuals’ self-rated emotional intelligence. Furthermore, in a study by Buhrmester et al. (1988), individuals’ self-rated interpersonal competence (for two out of five subscales) was related to the same ratings made by new acquaintances after brief interactions. In addition, there is some evidence that specific social skills rated as part of assessment centers are related to candidates’ personality self-concepts (e.g., *r* = .27 between extraversion and influencing others; Dilchert and Ones 2009; see also Collins et al. 2003; Spector et al. 2000). However, these relationships are challenging to interpret because most assessment centers do not reliably differentiate between the constructs they intend to measure (Breil et al. 2022a; Jackson et al. 2016; Kleinmann and Ingold 2019). Overall, there is a need for more robust insights into the relationships between expressions of social skills and self-concepts.

### 1.3. Differences between Social Skills

Depending on the interpersonal situation, different social skills are required to successfully handle the situation. For example, when someone needs to be convinced, individuals must show persuasiveness, whereas when someone is desperate, individuals need to show compassion. Therefore, it is not sufficient to assess only general “social competence,” but there is a need to differentiate between social skills. Here, it is best to select social skill dimensions that are clearly distinguishable at the behavioral level and can thus be reliably observed. Research from personality psychology has shown evidence of three distinct social skill dimensions. These include *agency* (i.e., getting ahead in social situations) and *communion* (i.e., getting along in social situations) as central behavioral differences in interpersonal interactions (Dawood et al. 2018; Wiggins 1979). Furthermore, recent studies have suggested that individuals also vary in the extent to which they can remain calm and relaxed when handling stressful interpersonal situations (*interpersonal resilience*; Breil et al. 2022a, 2022b; Hirschmüller et al. 2015; Leising and Bleidorn 2011).

As for the relationships between the expressions of these skill dimensions and the corresponding global self-concepts, it may well be the case that the magnitude varies depending on the social skill. However, for relationships between personality self-concepts and personality expression, the results have been mixed. That is, some studies have shown a lot of variability between traits (e.g., the relationship between expressed extraversion and the corresponding self-concept was much higher than for other traits; Borkenau et al. 2004), whereas in other studies, the differences were negligible (e.g., Back et al. 2009). Given the differences between maximum and typical performance, it is unclear so far how this relationship will translate to different social skills.

### 1.4. Present Research

In the current research, we examined how personality and skill self-concepts (i.e., assessed via questionnaires) are related to actual self-rated and observer-rated skill expression (i.e., assessed after participating in interpersonal exercises). Expressed skills were assessed as part of different interpersonal role-plays in which specific behavioral responses (i.e., skill expression) were needed to perform effectively. Based on previous research (Breil et al. 2022a, 2022b; Leising and Bleidorn 2011; Mielke et al. 2022), we focused on three different social skills: *agency* (i.e., getting ahead in social situations), *communion* (i.e., getting along in social situations), and *interpersonal resilience* (i.e., staying calm in social situations). In order to capture the skills as “purely” as possible, we created the role-plays specifically to assess only one skill. The three skills directly correspond to the three relevant Big Five dimensions (i.e., extraversion, agreeableness, negative emotionality) and the three relevant BESSI dimensions (i.e., social engagement, cooperation, emotional resilience), respectively. For an overview of which skill expression corresponds to which self-concept dimension, see Table 1.^2^

For each of the three skill dimensions, we analyzed the relationships between the expressed skills and the corresponding self-concepts as measured with personality or skill questionnaires. Thus, our research questions are as follows:

**Research** **Question** **1a** **(RQ1a).** *How are individual differences in observer-rated skill expression related to individual differences in corresponding personality and skill self-concepts?*

**Research** **Question** **1b** **(RQ1b).** *How are individual differences in self-rated skill expression related to individual differences in corresponding personality and skill self-concepts?*

Furthermore, because the BESSI questionnaire was specifically designed to measure skills, one could argue that individual differences in self-concepts assessed via the BESSI would be better suited for (i.e., would show incremental validity in) predicting actual skill expression compared with individual differences assessed via classic personality questionnaires.

Therefore, we also asked:

**Research** **Question** **2a** **(RQ2a).** *Are individual differences in observer-rated skill expression predicted by the corresponding skill self-concepts when controlling for the corresponding personality self-concepts?*

**Research** **Question** **2b** **(RQ2b).** *Are individual differences in self-rated skill expression predicted by the corresponding skill self-concepts when controlling for the corresponding personality self-concepts?*

Please note that all research questions, corresponding statistical analyses, and data preparation steps have been preregistered (see https://osf.io/h6dxe).

## 2. Materials and Methods

### 2.1. Participants

The initial sample consisted of 150 first-year medical school students who took part in a mandatory training course. Out of the initial 150 students, 137 (88 women; age: 17 to 31; *M* = 20.30, *SD* = 2.95)^3^ provided consent for their data to be analyzed for scientific purposes. Because this training course was a one-time event, the maximum possible sample size was limited to all first-year students who provided informed consent. Given our final sample size, we had a power of .80 to detect medium (i.e., *r* = .23) effect sizes for the main research question (i.e., Research Question 1). The university’s institutional review board approved this study (2017-28-GH-ÄA).

### 2.2. Procedures

This study was part of an actual training course for first-year medical students. After a brief introduction, the participants were asked to provide informed consent and to fill out two self-report questionnaires (i.e., the BFI-2 and BESSI) on tablets.

Afterward, they participated in three interpersonal role-play exercises. This procedure followed classic assessment or development centers, such that participants rotated through multiple standardized exercises and were observed by trained assessors. This specific procedure has been labeled “Multiple Speed Assessments” or “Multiple Mini Interviews” (in the medical community), highlighting that the exercises were relatively short (see Breil et al. 2020; Breil et al. 2022a; Herde and Lievens 2020, 2022; Knorr and Hissbach 2014). For each exercise, participants had 90 s to read short introductions and then walked into a room in which the exercise took place. Each exercise lasted for 5 min, and participants were specifically asked to try to achieve the goal of each exercise to the best of their ability. During all exercises, the participants interacted with professional actors who followed specific instructions regarding their roles and behavior. 

Each exercise revolved around one of three social skills (i.e., agency, communion, interpersonal resilience). That is, the exercises were designed so that individual differences in their respective skills would emerge. Each exercise was also designed to revolve as exclusively as possible around this one skill so that showing the skill was necessary to successfully complete the exercise. All exercises focused on everyday (nonmedical) tasks, and no prior knowledge was needed (see Table 2 for an overview). The three exercises used in this research have previously been validated in a high-stakes student selection context (Breil et al. 2022a).

While completing the exercises, the participants were generally observed by two observers (per participant, per exercise) through one-way mirrors.^4^ After each exercise, participants switched to the next exercise, and observers had to rate participants on the corresponding social skill dimension. All observers were undergraduate and graduate psychology students. They had been given extensive training (1.5 h per exercise), which included a small lecture as well as viewing and discussing example videos.

After completing all three exercises, participants were asked to rate how well they performed the respective skills as part of each specific exercise. Finally, participants were debriefed concerning this training course and were given general feedback. 

### 2.3. Measures

All measures, including the specific items, are described in detail in the study’s Codebook on https://osf.io/74qnu/. Furthermore, descriptive statistics (means, standard deviations, and reliabilities) of all measures are presented in Table 3, Table 4 and Table 5.

#### 2.3.1. Observer-Rated Skill Expression

Observers rated participants on one social skill per exercise via global, behaviorally anchored rating scales (e.g., please rate the participant’s expressed agency; the participant demonstrates assertive, confident, decisive, and energetic behavior; see Table 1). The specific behavioral anchors that helped to identify the degree of skill expression included a mixture of nonverbal and verbal behaviors (e.g., self-confident posture and clear statements for agency; see the codebook for details). The ratings were provided on a scale ranging from 1 (*low level of the respective skill*) to 6 (*high level of the respective skill*). Interrater agreement was excellent for all skills (see Table 3, Table 4 and Table 5).

#### 2.3.2. Self-Rated Skill Expression

Participants were also asked to provide their own evaluations of how well they managed to perform the skills corresponding to the exercises. That is, they were given the same rating items as the assessors (without the behavioral anchors) and were asked to indicate their skill level (on the same scale ranging from 1 to 6; see Table 1).^5^

#### 2.3.3. Personality Self-Concepts

Personality self-concepts were assessed using the German short version of the 30-item Big Five Inventory 2 (BFI-2-S; Rammstedt et al. 2020; Soto and John 2017). Participants were asked to indicate their level of agreement with each item on a scale ranging from 1 (*do not agree at all*) to 5 (*totally agree*). As part of this research, we used the Big Five dimensions of extraversion, agreeableness, and negative emotionality, as well as their facets.

#### 2.3.4. Skill Self-Concepts

The skill self-concepts were assessed with the German version of the Behavioral, Emotional, and Social Skills Inventory (BESSI; Lechner et al. 2022; Soto et al. 2022). Only skills relevant to the current research were assessed. These included leadership skill, persuasive skill, perspective-taking skill, capacity for social warmth, stress regulation, and anger management. These skills were chosen because they were most similar to the skills assessed in the exercises. Each skill was assessed with six items, and participants were asked how well they could perform an activity on a scale ranging from 1 (*not at all well*) to 5 (*extremely well*).

We further aggregated the corresponding skills from each dimension into one broader skill. That is, leadership skill and persuasive skill were aggregated into an agency skill self-concept, perspective-taking skill and capacity for social warmth were aggregated into a communion skill self-concept, and stress regulation and anger management were aggregated into an interpersonal resilience skill self-concept. This approach was supported by previous research on the BESSI, showing that conceptually related skills can be summarized into broader domains (Lechner et al. 2022; Soto et al. 2022). Additionally, the results of an exploratory factor analysis showed evidence for the proposed three domains (see Online Supplement S1 for scree plot and loadings).

### 2.4. Data Preparation and Analytic Strategy

There were a few cases in which we had missing data across measures (e.g., participants took part in the role-plays but did not fill out a specific questionnaire). For these cases, we used pairwise deletion.

For Research Question 1, we calculated Pearson correlations across the relevant variables. For Research Question 2, we computed a variety of multiple regressions. That is, observer-rated skill expression was regressed on the aggregated corresponding personality self-concept and skill self-concept. In additional models, we also investigated the relationships on the facet level (i.e., each specific skill self-concept was compared with the corresponding Big Five facet). Effect size interpretation was guided by recommendations for research on individual differences (Funder and Ozer 2019; Gignac and Szodorai 2016) with correlations of 0.10, 0.20, and 0.30 considered small, medium (typical), and relatively large. All statistical analyses were performed in R (for anonymized data and code, see https://osf.io/74qnu) and preregistered (see https://osf.io/h6dxe).

## 3. Results

### 3.1. Research Question 1

Research Question 1 addressed the relationships between self- and observer-rated skill expression and the corresponding personality and skill self-concepts. The results are presented in Table 3, Table 4 and Table 5. For observer-rated skill expression (RQ1a), we found large relationships between agency and the corresponding personality self-concept (i.e., extraversion; *r* = .36, *p* < .001) and skill self-concept (i.e., agency skill: *r* = .31, *p* < .001). That is, individuals with higher agentic self-concepts were also evaluated as more agentic in their actual behavioral expression. For communion and interpersonal resilience, there were no significant relationships with either personality (i.e., agreeableness: *r* = .10, *p* = .234; negative emotionality *r* = .00, *p* = .993) or skills (i.e., communion skill: *r* = .10, *p* = .246; interpersonal resilience skill: *r* = −.13, *p* = .135). This, in turn, shows that individuals with higher communion or interpersonal resilience self-concepts were not evaluated as more communal or resilient in their actual behavioral expressions.

For self-rated skill expression (RQ1b), we found medium to large effects across all three skills and for both the personality self-concepts (i.e., extraversion: *r* = .24, *p* = .005; agreeableness: *r* = .28, *p* = .001; negative emotionality *r* = −.17, *p* = .046) and the skill self-concepts (i.e., agency skill: *r* = .29, *p* < .001; communion skill: *r* = .49, *p* < .001; interpersonal resilience skill: *r* = .25, *p* = .004). This indicates, for example, that individuals who saw themselves as generally high in communion also evaluated their own behavior in a given situation as more communal. Of course, some of these high relationships may be driven by common method bias, given that both self-concepts and self-rated skill expression were assessed via similar self-report questionnaires.

Concerning the relationships between self- and observer-rated skill expressions, we found convergence for all skills. However, the effects were stronger for agency (*r* = .44, *p* < .001) than for communion (*r* = .22, *p* = .011) or interpersonal resilience (*r* = .24, *p* = .007). Furthermore, relationships across different skill expressions for one rating source were small to medium in size (i.e., observer-rated skills average intercorrelation = .18; self-rated skills average intercorrelation = .13). That is, individuals who excelled in the expression of the agency skill were not necessarily equally able to excel in the expression of the communion skill. The results also held when we controlled for age and gender.^6^

In additional (i.e., non-preregistered) analyses, we also investigated the relationships between self-concepts and non-corresponding skill expression (e.g., extraversion self-concept and expressed communion). For observer-rated skill expression, there were no significant results across skills. However, there was a significant relationship across skill domains for self-rated skill expression. For example, individuals with a high agentic skill self-concept or a lower negative emotionality self-concept indicated higher self-rated skill expression for all three skills (i.e., agency, communion, and interpersonal resilience; see Online Supplement S3 for all results). 

Finally, we estimated the relationships between self-concepts and corresponding skill expression in a latent variable framework. The relevant latent correlations were slightly higher than the manifest estimations but otherwise yielded no new insights (see Online Supplement S4).

### 3.2. Research Question 2

Research Question 2 dealt with the potential incremental validity of skill questionnaires above and beyond personality questionnaires in predicting social skill expression. For observer-rated expression (RQ2a), the skill self-concepts were not incrementally valid (see Table 6).^7^ On the contrary, for agency skill expression, the self-concept assessed with the personality questionnaire (i.e., BFI-2: extraversion) had incremental validity over the self-concepts assessed with the skill questionnaire (i.e., BESSI: agency skill, leadership skill, and persuasive skill; see Online Supplement S5).

For self-rated skill expression (RQ2b), we found diverging results. Here, the skill self-concepts showed incremental validity in predicting skill expression across all three skills (see Table 6). That is, given the same level of personality self-concept (e.g., extraversion: “I am someone who is dominant”), individuals with a higher skill self-concept (e.g., leadership: “I am good at taking charge of a situation”) also indicated higher self-viewed skill expression (e.g., agency skill expression: “I behaved assertively”). The results differed somewhat when considering skills at the facet level. For communion, both skill self-concepts (i.e., perspective taking and capacity for social warmth) were incrementally valid. However, for agency, only leadership was incrementally valid (but not persuasion), and for interpersonal resilience, only stress regulation was incrementally valid (but not anger management).

## 4. Discussion

In this study, we investigated how personality and skill self-concepts are related to individual differences in actual social skill expression. For this purpose, we used a behavioral-based approach to assess different social skills, which represents a departure from previous skill research that focused heavily on global self-assessments (Abrahams et al. 2019; Duckworth and Yeager 2015). Although all results should be interpreted with caution due to the relatively small sample size, this study provides initial evidence that both personality and skill self-concepts are related to self-rated skill expression and partly to observer-rated skill expression. For communion and interpersonal resilience, neither personality nor skill self-concept predicted observer-rated skill expression. However, there was a consistent and substantial association between self-reports and actual behavioral skill expression in the agency domain. That is, individuals’ reports on how extraverted they are, how strong their leadership and persuasive skills are, and how much they were able to display assertive, confident, decisive, and energetic behaviors were consistently and moderately to strongly related to how skillfully they indeed acted.

### 4.1. Implications for Theory and Practice

Our findings have multiple implications for theory and practice. First, the presented results provide further evidence that it is useful to distinguish between personality and skills at the questionnaire level. That is, similar to Soto et al.’s (2022) results, skill and personality ratings converged substantially but also captured unique information. In our research, this was evident in the fact that the skills assessed with the skill questionnaire (i.e., the BESSI) showed incremental validity in predicting self-rated skill expression.

Second, the results underline that, both in the theoretical discussion and especially in the practical use of skills, a clear distinction should be made between skills assessed via self-report questionnaires and skills assessed via behavioral observations. That is, whereas both personality and skill self-concepts predicted self-rated skill expression (i.e., how individuals evaluated their own behavior), they predicted observer-rated skill expression only for agency (i.e., how trained observers evaluated participants’ *agentic* behavior). Even though one would not have expected perfect correlations, it was surprising that observer-rated expressed communion or interpersonal resilience was not related to corresponding skills or personality self-concepts. This suggests that for some social skills, individuals’ self-view does not necessarily match the actual behavioral expression. That said, given the small sample size and the fact that skill expression was only examined in one (relatively short) situation, these results should be considered initial findings that need further replication.

Third, the relatively large differences across the three skills of agency, communion, and interpersonal resilience emphasize that it is important and necessary to differentiate between different social skills (see Soto et al. 2021). For example, the lack of predictive power for self-concepts applied primarily to communion and interpersonal resilience. That is, individuals do not seem to have accurate insights into how well they are able to get along with others or stay calm in stressful social situations. However, they did have knowledge about how well they are able to get ahead in social situations (i.e., agency). This finding is consistent with the general findings that self-concepts of extraversion or agency are much more strongly reflected in behavior than are the case for other traits (e.g., Borkenau et al. 2004; Breil et al. 2021). Another explanation for the differences is that the questionnaires for communion and interpersonal resilience also covered cognitive and emotional aspects that are not necessarily observable to others (e.g., communion: “feel compassion for other people”; interpersonal resilience: “stop myself from worrying”).

Fourth, the results also have implications for practice when training or selecting for social skills. Although self-rated and observer-rated expressed skills were related to a substantial degree (especially for agency), the relationship is far from perfect. This is striking since participants and observers rated the exact same situations and behaviors with the same behavioral anchors. This finding suggests that there might be remaining informational (e.g., one might not realize how awkward one’s behavior is) or motivational limits (e.g., one deliberately portrays oneself more favorably) for the participants. Informational limits should be considered when training social skills, e.g., by videotaping behavior or obtaining feedback from external observers. Motivational limits are especially important to consider in high-stakes situations, such as in personnel selection contexts. When using self-ratings, it is easy to present oneself in an overly positive way (Viswesvaran and Ones 1999). Therefore, if one wants to limit informational and motivational limits in selection settings, we suggest focusing on actual behavioral observation.

### 4.2. Limitations and Future Directions

A number of limitations that point to further research should be noted. First, as part of this study, we collected extensive behavioral data that provided us with initial insights into the relationships between self-concepts and behavioral expression. However, as the sample size was relatively small, future research should focus on obtaining more robust insights into the presented relationships.

Second, in this research, we purposefully designed the situations to elicit behavioral differences in specific social skills. This led to reliable and clearly distinguishable individual differences across the three skills (Breil et al. 2022a). However, the ratings for each skill were based on the evaluation of only one situation. This is a limitation because person effects become particularly stable as the number of situations increases (Epstein 1979). This principle of aggregation has repeatedly been shown in both laboratory (e.g., Borkenau et al. 2004; Funder and Colvin 1991; Leikas et al. 2012) and field studies (e.g., Fleeson and Gallagher 2009; Sherman et al. 2010; Sherman et al. 2015). For example, some individuals might not have been able to show their “true” skill levels due to specific situational aspects or other non-systematic influences. Such effects average out only across multiple situations. Thus, future research should investigate whether and how the predictive power of global self-concepts increases when assessing skill expression in multiple different situations.

Third, we focused on three distinguishable social skills that become visible in interpersonal situations. However, there are other relevant areas of skills from the “noncognitive” realm that were not assessed in this research. This includes, for example, self-management skills (e.g., time management) and innovation skills (e.g., abstract thinking skills), which are related to the Big Five dimensions of conscientiousness and openness, respectively (Soto et al. 2022). Although the assessment of such nonsocial skills was beyond the scope of the current study, it would certainly be fruitful to investigate how behavioral expressions of these skills are related to their self-concepts. 

Fourth, besides self-report questionnaires and self- and observer-rated skill expression, there are other methods or sources of information that can be used to infer individual differences in social skills. These include informant-reports (e.g., friends or family members filling out skill questionnaires; e.g., Buhrmester et al. 1988; Soto et al. 2022), Situational Judgment Tests, SJTs; (e.g., Lievens and Sackett 2012; Mielke et al. 2022), or specific socioemotional performance tests (i.e., tests that aim to assess emotional “intelligence,” theory of mind, cognitive empathy, or related aspects; e.g., Baron-Cohen et al. 1997; Conzelmann et al. 2013; Dziobek et al. 2006; Mayer et al. 2003). All of these methods have specific advantages and disadvantages. For example, SJTs allow for an economic assessment of individuals’ skill expression across a large number of hypothetical social situations; however, they are bounded by the limitations of self-reports (i.e., because they assess how individuals *believe* they would handle social situations). Socioemotional performance tests assess objectively measurable maximum performance differences; however, similar to questionnaires, many tests suffer from jingle-jangle fallacies, low reliabilities, and unclear relationships with actual behavioral expression (Brackett et al. 2006; Mota et al. 2019; Olderbak and Wilhelm 2020). Future research should compare these different assessment methods, as this would allow for an even more comprehensive understanding of social skills.

Fifth, this research does not provide any information about which way of assessing skills is “superior” to the other. This is an empirical question that future research should focus on using outcome criteria, such as success in education or work. Regardless of the potential incremental validity of any method, future research should also further discuss the conceptual core of social skills. Whereas for personality or emotional skills, it can certainly be argued that the self-concept incorporates additional relevant aspects of personality that are not necessarily reflected in behavioral expressions (e.g., individual differences in feeling, thinking, and desiring; see Back 2021; Breil et al. 2022c), it is questionable whether this argument also holds for social skills. That is, according to many definitions (Breil et al. 2022a; Kanning 2009a) social skills revolve around how one behaves in interpersonal situations and how this behavior in turn is perceived by others, regardless of one’s own feelings or desires.

## 5. Conclusions

The present study contributes to the understanding of individual differences in social skills. Specifically, we used a behavioral-based assessment approach in which participants interacted with professional actors across various short interpersonal simulations (one simulation per skill). Here, we investigated the extent to which questionnaires could predict such skill expression. We demonstrated that questionnaires were able to predict self-rated skill expression but not necessarily observer-rated skill expression. Actual, observer-rated skill expression converged with personality and skill self-concepts in the domain of agency but not in the domains of communion and interpersonal resilience. These findings highlight that it is necessary to differentiate between (a) personality and skills, (b) skill self-concepts and expressions of actual skills, and (c) different social skills (i.e., agency, communion, interpersonal resilience). 

## Figures and Tables

**Table 1:** Dimensions and Corresponding Skill Expression Ratings and Self-Concepts.

Dimension	Observer-Rated Skill Expression	Self-Rated Skill Expression	Personality Self-Concept (Assessed via the BFI-2 S)	Skill Self-Concept (Assessed via the BESSI)
Agency	This participant demonstrates assertive, confident, decisive, and energetic behavior *(1 item)*	How assertive, confident, decisive, and energetic was your behavior in this exercise? *(1 item)*	Extraversion Facets: sociability, assertiveness, energy level *(6 items, 2 per facet)*	Facets: Leadership skill *(6 items)* Persuasive skill *(6 items)*
Communion	This participant demonstrates warm, friendly, and compassionate behavior *(1 item)*	How warm, friendly, and compassionate was your behavior in this exercise? *(1 item)*	Agreeableness Facets: compassion, respectfulness, trust *(6 items, 2 per facet)*	Facets: Perspective-taking skill *(6 items)* Capacity for social warmth *(6 items)*
Interpersonal resilience	This participant demonstrates calm, relaxed, and emotionally balanced behavior *(1 item)*	How calm, relaxed, and emotionally balanced was your behavior in this exercise? *(1 item)*	Negative Emotionality Facets: anxiety, depression, emotional volatility *(6 items, 2 per facet)*	Facets: Stress regulation *(6 items)* Anger management *(6 items)*

*Note.* BFI-2 S = Big Five Inventory-2 short version; BESSI = Behavioral, Emotional, and Social Skills Inventory.

**Table 2:** Overview of Skill Dimensions and Exercises.

Dimension	Exercise
Agency	Persuasion: Participants had to pass on important information and convince someone to do something. The professional actor acted distracted and unconvinced.
Communion	Crisis: Participants had to take care of someone after a crisis situation and provide support. The professional actor acted overwhelmed and shocked.
Interpersonal resilience	Presentation: Participants had to give a presentation in front of someone but had little time to prepare. The professional actor acted unimpressed and cynical.

**Table 3:** Relationships of Skill Expression With Personality and Skill Self-Concepts: Agency.

	*M*	*SD*	Rel	1	2	3	4	5	6	7	8
1 Observer-rated skill expression: Agency	3.64	1.43	.85								
2 Self-rated skill expression: Agency	3.21	1.26	-	**.44**							
3 Self-concept: Extraversion	3.34	0.59	.86	**.36**	**.24**						
4 Sociability	3.00	0.79	.54	**.22**	**.18**	**.81**					
5 Assertiveness	3.35	0.74	.67	**.27**	**.19**	**.74**	**.39**				
6 Energy level	3.67	0.74	.71	**.34**	**.19**	**.77**	**.47**	**.33**			
7 Self-concept: Agency skill	3.17	0.58	.93	**.31**	**.29**	**.66**	**.43**	**.74**	**.39**		
8 Leadership skill	3.19	0.62	.92	**.36**	**.31**	**.69**	**.41**	**.75**	**.46**	**.90**	
9 Persuasive skill	3.15	0.66	.91	**.21**	**.22**	**.51**	**.38**	**.59**	**.20**	**.91**	**.65**

*Note*. *N* = 133 to 137, *M* = Mean, *SD* = Standard Deviation, Rel = Reliability (ICC [1,*k*] for observer-rated skill expression, McDonald’s Omega for self-concepts). Scale = 1 to 6 for skill expression, 1 to 5 for self-concepts. Significant correlations (*p* < .05) are in bold.

**Table 4:** Relationships of Skill Expression With Personality and Skill Self-Concepts: Communion.

	*M*	*SD*	Rel	1	2	3	4	5	6	7	8
1 Observer-rated skill expression: Communion	4.43	1.31	.90								
2 Self-rated skill expression: Communion	4.47	1.08	-	**.22**							
3 Self-concept: Agreeableness	3.97	0.54	.85	.10	**.28**						
4 Compassion	4.08	0.60	.29	.11	**.38**	**.77**					
5 Respectfulness	4.27	0.65	.64	.09	**.23**	**.83**	**.48**				
6 Trust	3.54	0.76	.33	.06	.09	**.84**	**.43**	**.54**			
7 Self-concept: Communion skill	3.68	0.51	.91	.10	**.49**	**.59**	**.55**	**.51**	**.41**		
8 Perspective-taking skill	3.79	0.57	.93	.04	**.46**	**.59**	**.63**	**.50**	**.35**	**.90**	
9 Capacity for social warmth	3.57	0.57	.87	.14	**.41**	**.48**	**.36**	**.42**	**.38**	**.90**	**.62**

*Note*. *N* = 133 to 137, *M* = Mean, *SD* = Standard Deviation, Rel = Reliability (ICC [1,*k*] for observer-rated skill expression, McDonald’s Omega for self-concepts). Scale = 1 to 6 for skill expression, 1 to 5 for self-concepts. Significant correlations (*p* < .05) are in bold.

**Table 5:** Relationships of Skill Expression With Personality and Skill Self-Concepts: Interpersonal Resilience.

	*M*	*SD*	Rel	1	2	3	4	5	6	7	8
1 Observer-rated skill expression: Interpersonal resilience	4.28	1.25	.89								
2 Self-rated skill expression: Interpersonal resilience	2.80	1.41	-	**.24**							
3 Self-concept: Negative emotionality	2.62	0.65	.85	.00	**−.17**						
4 Anxiety	3.13	0.85	.61	−.03	**−.25**	**.88**					
5 Depression	2.29	0.72	.58	.05	−.08	**.77**	**.54**				
6 Emotional volatility	2.45	0.80	.60	−.01	−.10	**.82**	**.61**	**.42**			
7 Self-concept: Interpersonal resilience skill	3.12	0.57	.91	−.13	**.25**	**−.68**	**−.59**	**−.51**	**−.58**		
8 Stress regulation	2.93	0.66	.91	−.10	**.30**	**−.75**	**−.70**	**−.59**	**−.56**	**.87**	
9 Anger management	3.32	0.64	.90	−.13	.13	**−.43**	**−.33**	**−.29**	**−.45**	**.86**	**.51**

*Note*. *N* = 130 to 137, *M* = Mean, *SD* = Standard Deviation, Rel = Reliability (ICC [1,*k*] for observer-rated skill expression, McDonald’s Omega for self-concepts). Scale = 1 to 6 for skill expression, 1 to 5 for self-concepts. Significant correlations (*p* < .05) are in bold.

**Table 6:** Incremental Validity of Skill Self-Concepts in Predicting Skill Expression When Controlling for Corresponding Personality Self-Concepts.

Dependent Variable	Independent Variables	Observer-Rated Skill Expression β [95% CI]	Self-Rated Skill Expression β [95% CI]
Skill expression: Agency	Self-concept: Agency skill (controlling for extraversion self-concept)	.12 [−.09, .33]	**.23** [.02, .45]
	Self-concept: Leadership skill (controlling for extraversion self-concept facets)	.24 [−.01, .50]	**.37** [.10, .63]
	Self-concept: Persuasiveness skill (controlling for extraversion self-concept facets)	.06 [−.14, .27]	.16 [−.05, .37]
Skill expression: Communion	Self-concept: Communion skill (controlling for agreeableness self-concept)	.06 [−.15, .27]	**.49** [.31, .68]
	Self-concept: Perspective-taking skill (controlling for agreeableness self-concept facets)	−.05 [−.28, .18]	**.36** [.16, .56]
	Self-concept: Capacity for social warmth (controlling for agreeableness self-concept facets)	.11 [−.08, .31]	**.36** [.19, .53]
Skill expression: Interpersonal resilience	Self-concept: Interpersonal resilience skill (controlling for negative emotionality self-concept)	**−.24** [−.48, −.01]	**.25** [.02, .47]
	Self-concept: Stress regulation (controlling for negative emotionality self-concept facets)	−.22 [−.48, .05]	**.35** [.10, .60]
	Self-concept: Anger management (controlling for negative emotionality self-concept facets)	−.17 [−.37, .03]	.10 [−.09, .29]

*Note.* The results refer to standardized effects. Significant βs (*p* < .05) are in bold.

## Data Availability

We have published all the raw data necessary to reproduce the reported results and provide scripts for all data analyses reported in this manuscript (see https://osf.io/74qnu).

## Notes

1 In this research, we use the term social skills to denote social, emotional, and behavioral skills or competencies (see Soto et al. 2021; Soto et al. 2022) that primarily take place in the interpersonal domain. In line with previous research (e.g., Schoon 2021) we use the terms skills and competencies interchangeably.

2 The remaining dimensions of the Big Five (consciousnesses, openness) and the BESSI (self-management, innovation) were deemed less “social” or “interpersonal,” as their emergence is most likely independent of the social partner. The skill dimension revolving around emotional resilience or negative emotionality is also not “purely” interpersonal. Here, however, we refer to how one deals with stressors (i.e., criticism, time pressure, negative feedback) in the *interpersonal* domain, and thus label this skill dimension “interpersonal resilience.” In fact, research has suggested, “Emotional Stability may be more interpersonal than is often assumed” (Leising and Bleidorn 2011, p. 990), and it has been shown that differences in emotional stability can be reliably assessed as part of (high-stakes) interpersonal situations (Breil et al. 2022b; Hirschmüller et al. 2015).

3 As our sample was predominantly female and quite homogeneous in terms of age and educational background, the data might be prone to range restrictions or floor and ceiling effects. In order to get a deeper understanding of our sample, we checked whether our data differ significantly from data reported in the original validation studies of the respective questionnaires (Lechner et al. 2022; Rammstedt et al. 2020). Our sample reported significantly higher values in all facets of extraversion and agreeableness in perspective-taking and anger management, and significantly lower values in depression, emotional volatility, and stress regulation. That is, for most self-concept measures, our sample tended toward the scale endpoints and also showed lower variance for all variables except for two BFI facets (i.e., trust and anxiety). As a result, the findings should be considered conservative estimates.

4 There were 24 observers in total. Eight observers were divided into teams of two and assigned to one exercise. This was the case because each exercise took place in parallel across the four rooms. Thus, each observer team evaluated roughly one-fourth of all participants. In about 25% of the cases, the second observer was not available. In these cases, we used only the ratings of the first observer. In addition, there were two participants whose behavior in the interpersonal resilience exercise was evaluated by three observers.

5 Participants were also asked to indicate how well they performed skills that were not directly assessed in the respective exercises (e.g., agency in the exercise that assessed communion). These answers were not used in the current project.

6 There was one exception to this: the relationship between self-rated interpersonal resilience and negative emotionality self-concept was no longer significant when we controlled for participants’ gender and age (*β* = −.12; *p* = .171). Self-rated interpersonal resilience expression was also the only case in which men and women significantly differed in their skill expression (i.e., men scored higher; Cohen’s *d* = 0.45, *p* = .017). There were no significant relationships between age and skill expression. For an overview of all gender and age differences in self- and observer-rated skill expression, see Online Supplement S2.

7 Interpersonal resilience was incrementally valid but in an unexpected direction. That is, when we controlled for negative emotionality self-concept, individuals with a higher interpersonal resilience self-concept were given lower skill expression ratings by observers.

## Supplementary Materials

The following supporting information can be downloaded at: https://osf.io/74qnu, Codebook, Online Supplement S1 to S5, Data, R code.
